# Advances in the study of HSP70 inhibitors to enhance the sensitivity of tumor cells to radiotherapy

**DOI:** 10.3389/fcell.2022.942828

**Published:** 2022-08-10

**Authors:** Sihan Du, Ying Liu, Yuan Yuan, Yuran Wang, Yanfang Chen, Shuai Wang, Yuhua Chi

**Affiliations:** ^1^ School of Medical Imaging, Weifang Medical University, Weifang, Shandong, China; ^2^ Department of Radiotherapy, Affiliated Hospital of Weifang Medical University, Weifang, Shandong, China; ^3^ Department of General Medicine, Affiliated Hospital of Weifang Medical University, Weifang, Shandong, China

**Keywords:** HSP70, HSP70 inhibitor, radiotherapy, sensitivity, tumour

## Abstract

The 70 kDa heat shock protein (HSP70) is one of the most conserved proteins and a ubiquitous molecular chaperone that plays a role in the folding, remodeling, and degradation of various proteins to maintain proteostasis. It has been shown that HSP70 is abundantly expressed in cancer and enhances tumor resistance to radiotherapy by inhibiting multiple apoptotic pathways, such as interfering with the cellular senescence program, promoting angiogenesis, and supporting metastasis. Thus, HSP70 provides an effective target for enhancing the effects of radiation therapy in the clinical management of cancer patients. Inhibition of HSP70 enhances the radiation-induced tumor-killing effect and thus improves the efficacy of radiotherapy. This article reviews the sensitivity of Hsp70 and its related inhibitors to radiotherapy of tumor cells.

## Introduction

The Heat shock proteins (HSPs) family, highly conserved, are present in all organisms and play a vital role in protein folding, translocation and degradation, hence their name molecular chaperones, a concept first used by Laskey et al. (1978). They were first discovered in 1962, when Ritossa and colleagues observed that the giant chromosomes contained heat-inducible genes and heat-inducible proteins in the gland cells of Drosophila larvae exposed to high temperatures, opening a new field of study of heat shock responses (Ritossa, 1962). HSPs are classified into several families such as HSPH (HSP110), HSPC (HSP90), HSPA (HSP70), HSPD (HSP60), DNAJ (HSP40), and HSPB (small HSPs) (Kampinga et al., 2009). Among them, Hsp70 is the most conserved protein in evolution, which can stabilize proteins against denaturation, help nascent proteins to fold, and dissolve damaged or abnormal proteins. Hsp70 is not only produced under heat stress but also expressed under stress conditions such as heavy metals, small molecule chemical toxins, infection, oxidative stress, inflammation, ischemia, cancer, and neurodegenerative diseases, among other stress conditions (Westerheide and Morimoto, 2005). Notably, tumors frequently overexpress HSP70 in the cytoplasm, present HSP70 on their plasma membrane, and actively release HSP70 compared to normal cells (Multhoff et al., 2015). And it has been demonstrated that HSP70 is associated with early tumor recurrence, poor prognosis, and resistance to radiotherapy (Brondani Da Rocha et al., 2004; Yang et al., 2015; Jin et al., 2016).

Hsp70 is closely related to tumor therapy such as tumor radiotherapy and chemotherapy, among which radiotherapy is a commonly used tumor treatment method. After prolonged radiation exposure of tumor cells in radiotherapy, some genes and proteins are expressed abnormally so that tumor cells acquire a radiation-resistant phenotype and become less radiosensitive, leading to increased invasion, metastasis, and recurrence of certain tumors (Kim et al., 2015). Therefore, many researchers have worked to discover a radiosensitizer with good pharmacological properties to enhance the sensitivity of tumor cells to radiotherapy and thus improve the efficacy of radiotherapy. Studies have shown that HSP70 plays an essential role in tumor transformation and tumor development, as they are both necessary for tumor cell survival and growth and are closely related to tumor radioresistance (Table 1). Moreover, malignant cells express higher levels of HSP70 than normal cells, and high expression of HSP70 induces resistance to radiotherapy (Xu et al., 2010; Kumar et al., 2016). Inhibition of HSP70 inhibits the growth of many tumor cells and significantly promotes radiation-induced apoptosis, increasing the sensitivity of tumors to radiotherapy. Thus, HSP70 inhibitors can be used as radiosensitizers to enhance the efficacy of radiotherapy (Wen et al., 2014). HSP70 inhibitors can be classified as small molecules or compounds. Common small molecule-like inhibitors include 2-phenylethynesulfonamide (PES) and Apoptozole (Az), a small molecule inhibitor of HSP70 ATPase activity (Leu et al., 2011; Yang et al., 2020a), and common compound-like inhibitors such as the naturally occurring flavonoids -Epigallocatechin gallate (EGCG) and quercetin (Li et al., 2009).

**TABLE 1:** The role of HSP70 in resistance to radiotherapy.

Cancer type	Findings	References
Lung cancer	Induction of HSP70 expression and AKT phosphorylation by Redd1 to acquire resistance to radiotherapy	Jin et al. (2019)
Nasopharyngeal carcinoma	TPL (HSP70 inhibitor) enhances cellular radiosensitivity by inhibiting GRP78 to trigger apoptosis and induce G2/M phase arrest	Li et al. (2016)
Endometrial cancer	Silencing of HSP70 expression enhances radiotherapy efficacy	Du et al. (2009)
Oropharyngeal cancer	GRP78 (HSP70) confers radioresistance by increasing radiation-induced DNA double-strand break repair and cell autophagy and the subsequent inhibition of apoptosis	Sun et al. (2017)
Glioblastoma and lung cancer	Antibodies targeting GRP78 exhibit antitumor activity and enhance the efficacy of radiotherapy on tumors	Dadey et al. (2017)

TPL, triptolide.

Studies have found that inhibiting HSP70 (GRP78) can induce endoplasmic reticulum (ER) stress and trigger unfolded protein response (UPR), thereby inducing cancer cell apoptosis and slowing tumor growth (Jiang et al., 2007; Samanta et al., 2021). Jäättelä et al. (Nylandsted et al., 2000) first showed that silencing HSP70 by antisense RNA induced massive cell death in breast cancer cell lines but was not toxic to non-tumorigenic breast epithelial cells or normal human fibroblasts (Nylandsted et al., 2000), suggesting that targets HSP70 selectively induces tumor cell death. In addition, it has been shown that silencing HSP70 alone in the absence of HSC70 is toxic to some cancer cell lines (human cervical cancer, human breast cancer, human prostate cancer, and human liver cancer) but has no effect on normal cells (Rohde et al., 2005). Interestingly, however, in prostate cancer cells, only simultaneous silencing of HSP70 and HSC70 was accompanied by an increase in the rate of apoptosis (Wang et al., 2014). Workman et al. (Powers et al., 2008) reported a similar finding that only simultaneous silencing of HSP70 and HSC70 reduced cell survival in HCT116 colorectal cancer cells and A2780 ovarian cancer cells. Therefore, there may be cell-specific differences in sensitivity to HSP70 inhibition.

## HSP70 as a target for cancer therapy

**FIGURE 1:**
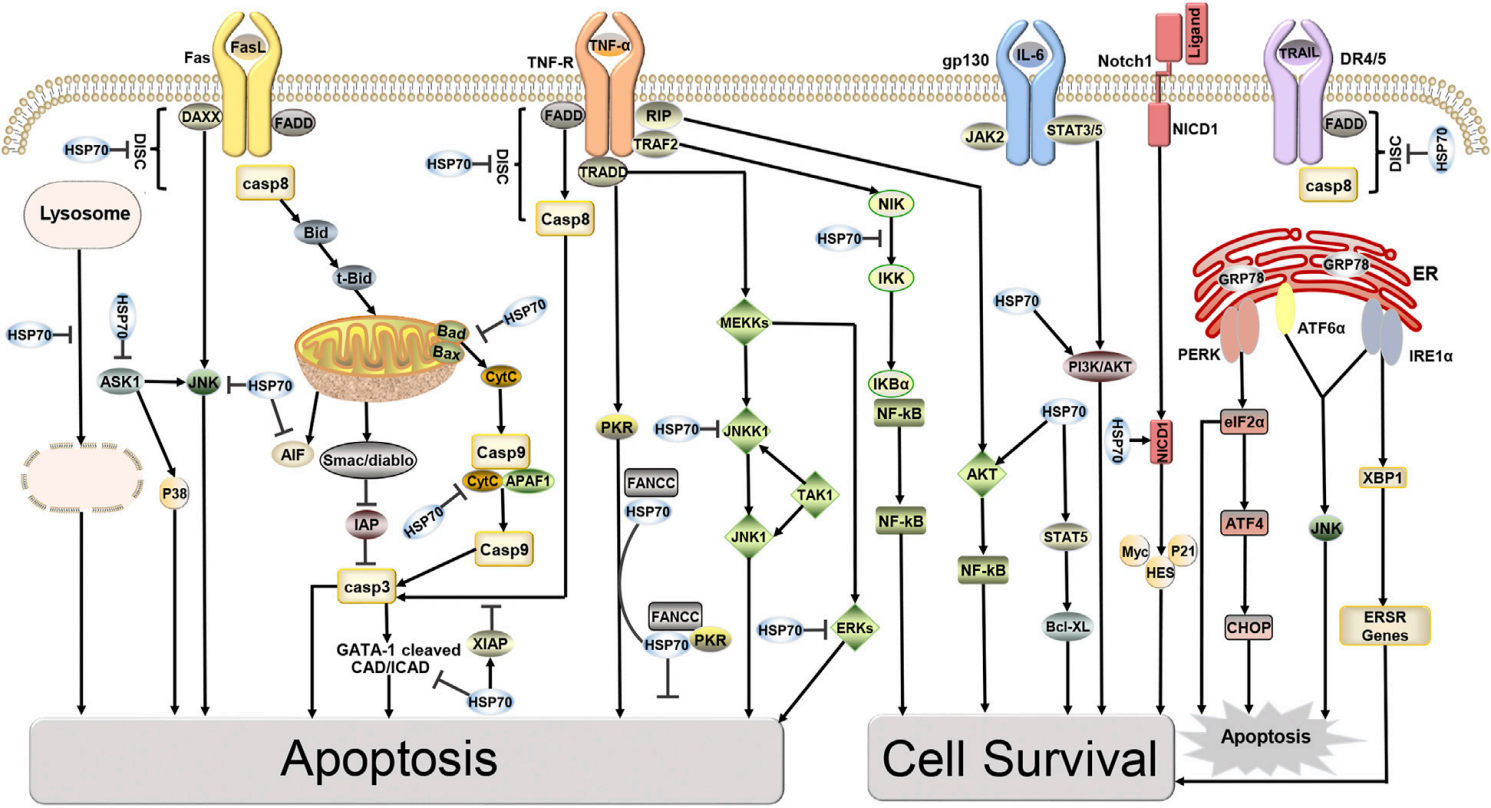
Targets of HSP70 in Apoptosis and Survival Pathways. HSP70 inhibits apoptosis, induces cell survival, and increases tumor resistance to radiotherapy through various pathways, including the mitochondrial pathway, death receptor pathway, DNA degradation pathway, and endoplasmic reticulum stress pathway.

Hsp70 is an important molecular chaperone and is abundantly expressed in malignancies of many different origins. Hsp70 not only inhibits apoptosis by reducing cytoplasmic Ca2+, stabilizing lysosomes, and inhibiting cellular protein aggregation but also forms stable complexes with tumor antigens in the cytoplasm, which can escape from dying cells intact and interact with antigen presenting cells (APCs) to suppress tumor immunity (Calderwood et al., 2005; Dudeja et al., 2009). In addition, overexpression of HSP70 induces the expression of MMP-9, which increases the invasiveness of tumor cells (Du et al., 2009). It is not surprising that HSP70 interacts with a variety of tumor-related proteins, as its binding sites are present in almost all protein sequences in the cell (Rüdiger et al., 1997). HSP70 is important as a molecular chaperone for protein folding, however, this is likely not its direct role in the cell death pathway. Beere et al. (2000) first suggested in 2000 that HSP70 could inhibit apoptosis by directly binding specifically to Apaf-1 to block the assembly of the apoptotic complex, but it could not block Fas-induced apoptosis. A subsequent study by Clemons overturned this conclusion that HSP72 acts upstream of mitochondria to inhibit Fas-mediated apoptosis and provide long-term cytoprotection in type II CCRF-CEM cells (Clemons et al., 2005). Furthermore, the discovery that HSP70 inhibits cell senescence establishes a clear link with cancer, and HSP70 controls the expression of major regulators of oncogene induced senescence (OIS). Knockdown of HSP70 in certain tumor epithelial cell lines leads to senescence, which provides a defense against cancer (Colvin et al., 2014; Sherman and Gabai, 2015). More importantly, overexpression of HSP70 provides a survival advantage for cancer cells as it can interact with multiple components of the caspase dependent and non-caspase-independent apoptotic pathway to inhibit cancer cell apoptosis (Figure 1). Mitochondrial pathway: In the pre-mitochondrial phase HSP70 reduces the permeability of the outer mitochondrial membrane by blocking the migration of Bax and Bad, thereby inhibiting the release of apoptotic factors such as cytochrome C (Cytc), apoptosis Inducing Factor (AIF), and SMAC/Diablo from the mitochondria (Vostakolaei et al., 2021). In late mitochondrial stages, HSP70 binds directly to apoptotic protease activating factor-1 (Apaf-1), blocking the aggregation of procaspase-9 so that the apoptotic complex cannot form and downstream caspase-3 cannot be activated (Saleh et al., 2000). An ultimate target of caspase-3 is the transcription factor GATA-1, however, HSP70 protects GATA-1 from cleavage by caspase-3, thereby increasing erythroid differentiation and survival (Dong et al., 2020). Death receptor pathway: HSP70 interacts with death receptors (TNFR, FasR, DR4/5) to prevent the formation of the death inducing signaling complex (DISC) and inhibits receptor-mediated stress kinases such as apoptosis signal-regulating kinase 1 (ASK1) and C-Jun amino acid terminal kinase 1 (JNK1) activation (Vostakolaei et al., 2021). The role of HSP70 in regulating Nuclear factor kappa B (NF-κB) function is controversial. It is hypothesized that HSP70 in the cytoplasm can repress NF-κB expression, whereas membrane-bound HSP70 can induce this transcription factor (Boudesco et al., 2018). HSP70 in the cytoplasm blocks NF-κB activation by inhibiting I-kB-α kinase (IKK) and degrading I-kB-α (Sheppard et al., 2014). In addition, in hematopoietic cells, TNFα activates pro-apoptotic double-stranded RNA-dependent protein kinase (PKR). One inhibitor of PKR is the Fanconi Anemia Complementation group C gene product (FANCC), in which HSP70 interacts with FANCC proteins through its ATPase structural domain to form the ternary complex HSP70, FANCC, and PKR to inhibit TNF-induced apoptosis (Pang et al., 2002). DNA degradation pathway: The HSP70, HSP40,and ICAD complex inhibit the activity and folding effect of Caspase Activated DNAse (CAD), preventing the degradation of chromosomal DNA at late stages of apoptosis, thus achieving an anti-apoptotic effect (Kumar et al., 2016). Endoplasmic reticulum stress pathway: The multiple environmental stresses experienced by tumor cells during growth, infiltration, and metastasis, such as ischemia and hypoxia, can cause endoplasmic reticulum stress to tumor cells. Endoplasmic reticulum stress initiates the unfolded protein response (UPR) to restore cellular homeostasis but mediates apoptosis when endoplasmic reticulum stress exceeds the cell’s ability to survive (Gong et al., 2017). When cells are exposed to endoplasmic reticulum stress, GRP78 (HSP70) binds to unfolded proteins and dissociates from protein kinase RNA-like ER kinase (PERK), inositol-requiring enzyme 1α (IRE1α) and activating transcription factor 6 (ATF6), and three signaling pathways are activated. Among them, IRE1 is activated by autophosphorylation, and activated IRE1 has RNase activity, which in turn shears 26 introns from XBP1 mRNA to generate active transcription factors XBP1s that promote tumorigenesis, cancer cell survival, and regulate tumor invasion (Nath et al., 2021). Other pathways: HSP70 promotes cell survival by activating PI3K/AKT, a downstream target of JAK2/STAT5 (De Almeida et al., 2020). Overexpression of HSP70 can upregulate the level and activity of STAT5, and phosphorylated STAT5 binds DNA and increases the expression of the anti-apoptotic protein Bcl-xL (Guo et al., 2005). HSP70 promotes tumor cell survival by inhibiting lysosomal membrane permeability (Nylandsted et al., 2004). In addition, HSP70 interacts with the Notch1 intracellular domain (NICD) and contributes to the activity of the cellular Notch signaling pathway (Juryńczyk et al., 2015). The above data suggest that HSP70 promotes tumor cell survival and inhibits tumor cell apoptosis in several ways. Inhibition of HSP70 can induce tumor apoptosis and increase radiosensitivity, providing evidence for Hsp70 as an anticancer target (Du et al., 2009).

**FIGURE 2:**
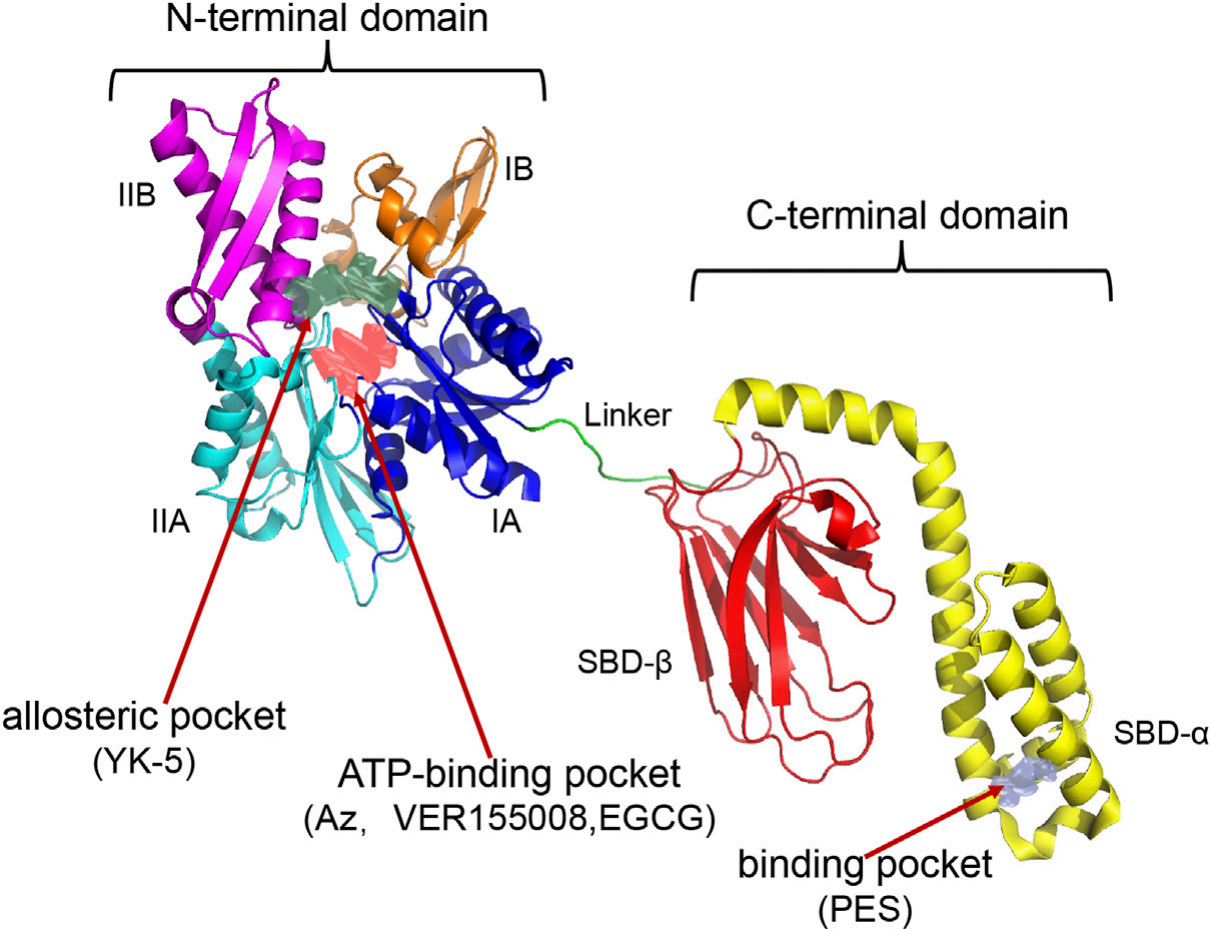
Establishment of HSP70 Homology Model and Potential Drug Sites. The following HSP70 inhibitors bind in separate pockets of HSP70: Az, VER155008, EGCG binds in the N-terminal ATP-binding pocket; YK5 binds in the N-terminal allosteric pocket (the allosteric pocket shown at the N-terminus in the figure is the binding site for YK-5); PES binds in the binding pocket of the C-terminal helical domain. The PDB code used to construct the homology model crystal structure is 1S3X, 2KHO.

HSP70 consists of an N-terminal nucleotide binding domain (NBD), a C-terminal substrate binding domain (SBD), and a linker connecting the N-terminal to the C-terminal. The N terminus comprises two adjacent lobes (lobe I and lobe II), each of which is further divided into two structural subregions (IA, IIA, IB, and IIB). The C-terminus consists of a twisted β-sandwich domain (SBDβ) that binds to the substrate and an α-helical lid domain (SBDα) that closes the substrate. HSP70 inhibitors can inhibit HSP70 activity by different mechanisms (Figure 2; Table 2), most known HSP70 inhibitors bind to the NBD, such as Az, VER155008, and EGCG which usually bind to the N-terminal ATP-binding pocket of HSP70 to inhibit HSP70 activity (Williamson et al., 2009; Tran et al., 2010; Cho et al., 2011). YK-5 interacts with the allosteric pocket of HSP70, a site located in the cleft region outside the ATP/ADP binding domain, flanked by subregions IB and IIB (Rodina et al., 2013); MKT-077 binds to an allosteric site within the nucleotide-binding domain (Rousaki et al., 2011); the HSP70 inhibitors 2,5′-thiodipyrimidine and 5-(phenylthio)-pyrimidine acrylamides designed by G. Chisosis et al. bound to a novel variant pocket of the NBD of HSP70 (Kang et al., 2014); HSP70-Bim protein-protein interaction (PPI) inhibitor, S1g-2, which is located in the hydrophobic cleft between the IA and IIA substructural domains of the Hsc70 NBD, induces cell-specific apoptosis in chronic myeloid leukemia (CML) cells by selectively disrupting HSP70-Bim PPI (Song et al., 2021). Some HSP70 inhibitors act by different mechanisms: 15-deoxyspergualin (DSG) inhibits HSP70 ATPase activity by interacting with the C-terminal EEVD motif (Nadler et al., 1998), and 2-phenylethynesulfonamide (PES) acts on the binding pocket in the C-terminal helical domain of HSP70 to inhibit its activity (Balaburski et al., 2013). A number of new HSP70 inhibitors have now been tested in preclinical models and they have been shown to increase the sensitivity of tumors to chemotherapy and radiotherapy (Multhoff et al., 2015). Here we mainly summarise the studies related to HSP70 inhibitors increasing tumor radiosensitivity.

**TABLE 2:** Demonstrates Hsp70 inhibitors including their sites of action and applications in preclinical studies and clinical trials.

Hsp70 inhibitors	Site of interaction	Preclinical studies	Clinical trials	Mechanism
MKT-077 (Rousaki et al., 2011; Wadhwa et al., 2000; Deocaris et al., 2007)	NBD	*In vitro* and *in vivo*	Phase I failed	Induces tumor growth arrest; induces senescence in human tumor cell lines
VER-155008 (Wen et al., 2014; Williamson et al., 2009; Sakai et al., 2021; Schlecht et al., 2013)	NBD	*In vitro* and *in vivo*		Inhibits tumour cell proliferation; inhibits cell cycle progression; promotes radiation-induced cancer cell death; disrupts signalling pathways
EGCG (Tran et al., 2010; Khan and Mukhtar, 2010; Ermakova et al., 2006)	NBD	*In vitro* and *in vivo*		Inhibits tumour cell proliferation; induces apoptosis; induces cell cycle arrest; inhibits tumour angiogenesis
Apoptozole (Cho et al., 2011; Ko et al., 2015; Park et al., 2017; Park et al., 2018)	NBD	*In vitro* and *in vivo*		Promotes lysosome-mediated apoptosis in tumour cells; impairs autophagy in cancer cells; inhibits tumour growth
YK-5 (Rodina et al., 2013)	NBD	*In vitro*		interferes with the formation of a competent chaperone/onco-client complex; induces cancer cell growth arrest; induces tumour apoptosis
JG-98 (Li et al., 2015; Yaglom et al., 2018)	NBD HSP70-BAG3 interaction	*In vitro* and *in vivo*		Affects cell signaling pathways; inhibits tumor growth; anti-proliferative; increases cell cycle-dependent kinase inhibitor P27, P21 levels; upregulates DNA damage response and UPR in tumor cells
S1g-2 (Song et al., 2021; Zhang et al., 2022)	NBD HSP70-Bim PPI	*In vitro* and *in vivo*		Interferes with signalling pathways; induces apoptosis in cancer cells
PES (Kumar et al., 2016; Jiang and Xiao, 2020; Leu et al., 2009)	SBD	*In vitro* and *in vivo*		Interferes with cell survival and signaling pathways; causes dysfunctional autophagy and altered lysosomal function; disrupts HSP70-associate chaperone interactions; induces cell cycle arrest; reduces HSP70 client protein levels; induces apoptosis in cancer cells
15-DSG (Nadler et al., 1998; Kawada et al., 2000; Kawada et al., 2002; Ramya et al., 2006)	C-terminal EEVD motif D	*In vitro* and *in vivo*	Phase II failed	Inhibits protein synthesis; induces apoptosis; inhibits tumor cell growth; inhibits tumor-forming angiogenesis; induces cell cycle arrest

EGCG, epigallocatechin-3-gallate; PES, 2-phenylethynesulfonamide; 15-DSG, 15-deoxyspergualin; UPR, unfolded protein response.

## Mechanisms of radiosensitization by HSP70 inhibitors

The protective effect of HSP70 against radiation-induced apoptosis may underlie resistance to tumor radiotherapy. During ionizing radiation-induced cell damage HSP70 is released into the tumor microenvironment, triggering the immune system to activate dendritic cell maturation and cytotoxic T cell recruitment, thereby inhibiting tumor cell apoptosis, increasing cancer cell resistance to ionizing radiation-induced oxidative stress, and inducing resistance to radiotherapy (Ashrafizadeh et al., 2020). HSP70 knockout mice exhibit not only increased susceptibility to necrosis and inflammation, but also genomic instability and enhanced radiosensitivity (Hunt et al., 2004). Therefore, Hsp70 inhibitors could be a promising candidate for cancer therapy to enhance the effects of clinical radiosensitization. The mechanism of radiosensitization by Hsp70 inhibitors is described here.

### Disruption of the activity of HSP70 chaperone clients

At the molecular level, the increased radiosensitivity of tumor cells induced by inhibition of HSP70 activity is closely associated with the down-regulation of protein expression of bcl-2, bcl-xl, mdr1, chk-1, and mcl-1 and the up-regulation of protein expression of bax, p53, fax and bad (Kim et al., 2013; Meng et al., 2014; Wang et al., 2020a; Tang et al., 2020). HSP70 inhibitors have been found to inhibit HSP70 activity in certain tumors by disrupting the activity of client proteins associated with HSP70, thereby inhibiting cell proliferation, inducing apoptosis and increasing tumor radiosensitivity (Li et al., 2013; Jiang and Xiao, 2020). Li et al. (2013) treated MDA-MB-231 and MCF-7 breast cancer cells with the HSP70 inhibitor MKT-077 analogue (JG-83) for 24 h and then assayed Akt1 and Raf1 by Western blot and found that the levels of Akt1 and Raf1 were reduced by approximately 25% in MDA-MB-231 and MCF-7 cells. This reflects the fact that HSP70 inhibitors modestly destabilize the chaperone clients Akt1 and Raf1 and induce apoptosis in these cells. Another study reached similar conclusions in oral squamous carcinoma cells, the HSP70 inhibitor PES reduced its client protein X-linked inhibitor of apoptosis protein (XIAP) and cellular inhibitors of apoptosis proteins 1 (c-IAP1) levels and significantly blocked the association of HSP70 with XIAP, suggesting that PES inhibited the function of HSP70 in oral squamous cell carcinoma cells by destroying HSP70 client proteins, and ultimately inhibiting the growth of oral squamous cell carcinoma cells (Jiang and Xiao, 2020).

In addition, inhibitors can target not only HSP70 but also the dimeric structure of HSP70 to promote apoptosis in tumor cells. Dimerization of HSP70 is essential for regulating HSP70 activity (Takakuwa et al., 2019; Pan et al., 2021), and common co-chaperone proteins of HSP70 such as Bim and JDP can combine with HSP70 as a dimer structure to regulate HSP70 activity and substrate specificity (Pan et al., 2021; Wyszkowski et al., 2021). In contrast, the HSP70-Bim PPI inhibitor S1g induces apoptosis in tumor cells by dissociating the HSP70/Bim dimer (Song et al., 2021; Wang et al., 2021). Among them, the co-chaperone Bim helps HSP70 recognize some cancer-associated clients, making some cancer cells such as T47D cells, BV173 cells, KCL-22 cells, etc. dependent on HSP70-Bim function (Guo et al., 2020). HSP70-bim PPI inhibits apoptosis by regulating the folding of oncogenic clients AKT and Raf-1 in cancer cells (Guo et al., 2020), whereas S1g-6, an HSP70 inhibitor targeting the tumor-associated HSP70-Bim PPI, significantly decreased the expression and phosphorylation levels of AKT and Raf-1 proteins when BV173 and KCL22 cells in CML cell lines were treated with S1g-6 (Wang et al., 2021). This data suggests that S1g-6 can disrupt HSP70-Bim function in living cells and has an anti-tumor effect. Several other studies have supported the idea that HSP70 inhibitors can increase the sensitivity of tumors to radiotherapy by modulating client proteins (Kim et al., 2014; Meng et al., 2014).

### Blocking the HSP70-dependent signalling pathway

Several studies have demonstrated that HSP70 inhibitors can promote apoptosis and increase tumor sensitivity to radiotherapy by blocking HSP70-dependent signaling pathways. For example, quercetin enhances the radiosensitivity of colon cancer cells *in vivo* by targeting Notch-1 signaling and colonic CSCs (Li et al., 2020). These authors found that quercetin combined with IR eliminated CRC stem cells (CRC-SCs) by inhibiting Notch1 signaling and that the expression of biomarkers in CSCs was significantly reduced in both *in vitro* and *in vivo* studies. Quercetin and IR inhibited the growth of primary and secondary colonosphere and suppressed the Notch-1 signaling pathway by downregulating γ-secretase complex proteins, Jagged-1, HES1, and HEY1. The above results suggest that the combination of quercetin and IR may be a promising strategy for tumor sensitization by radiotherapy. The PI3K/Akt signaling pathway plays a key role in the development of many tumors, and the activation of the PI3K/Akt pathway is closely associated with the chemoresistance and radioresistance of tumor cells (Fresno Vara et al., 2004). The PI3K/Akt signaling pathway is aberrantly activated in most malignancies and activated AKT phosphorylates downstream target proteins, including FOXO1, WEE1, GSK3b, and mTORC1, which lead to tumor cell survival, promote cell cycle progression, ribosome biogenesis or protein synthesis (Song et al., 2019). Therefore, the topic of whether HSP70 inhibitors can improve the radiosensitivity of tumor cells by inhibiting the PI3K/Akt signaling pathway is worthy of further investigation. Shu et al. (2021) demonstrated the possibility that TPL could radiosensitize U251 cells by inhibiting the PI3K/Akt signaling pathway. When the 10nmol/L HSP70 inhibitor triptolide (TPL) was combined with 4GyX-rays, the expression levels of p-PI3K and p-Akt in human glioma U251 cells decreased more significantly than with TPL or X-rays alone. In addition, CHOP is a transcription factor that promotes apoptosis by regulating apoptosis-related genes such as down-regulation of bcl2, bcl-xl, and mcl-1 expression and up-regulation of BIM, BAK, and Bax expression (Hu et al., 2018). Using a human ovarian cancer xenograft model, one study showed that quercetin induced CHOP via the PERK/ATF4/eIF2α pathway, thereby enhancing ionizing radiation-induced apoptosis (Gong et al., 2018). AKT, MAPK, and ERK signaling pathways are also important pathways that regulate cell cycle progression and apoptosis. PES inhibited the activation of Akt and ERK pathways to promote apoptosis in NSCLC cells (Zhou et al., 2017a) or induced apoptosis in oral squamous cell carcinoma cells by reducing the expression and phosphorylation levels of AKT and ERK (Jiang and Xiao, 2020), these data reflect the positive effects of PES in inhibiting tumor development. NF-κB is an important transcriptional activator involved in various physiological and pathological processes such as inflammatory response, immune response, and cell proliferation and apoptosis. NF-κB not only promotes cell proliferation and inhibits apoptosis, but upregulation of NF-κB in tumor cells also promotes upregulation of tumor metastasis factors and lymphatic barrier breach, which plays an important role in the development of tumorigenesis (Nguyen et al., 2015). Upregulation of NF-κB leads to inflammatory responses and oxidative stress, and it can lead to tumor resistance to radiotherapy and chemotherapy by regulating angiogenesis and inhibiting apoptosis (Mortezaee et al., 2019). Zhang et al. (2012) found that EGCG significantly enhanced radiation-induced apoptosis and was accompanied by a decrease in NF-κB protein levels, suggesting that HSP70 inhibitors may reduce tumor resistance to radiotherapy by inhibiting the NF-κB signaling pathway. In addition, Meng et al. (2014) reported a similar finding that the HSP70 inhibitor TPL could inhibit NF-κB activity not only directly by inhibiting the trans-activation of the p65 subunit of NF-κB but also indirectly through the AKT/GSK3β/mTOR pathway to inhibit NF-κB signaling and promote apoptosis. In summary, HSP70 inhibitors increase radiation-induced apoptosis in tumor cells by blocking a variety of HSP70-dependent signaling pathways.

### Inhibition of repair of radiation-induced DNA damage

DNA is the main target of ionizing radiation, which has cytotoxic effects on DNA, mainly resulting in single-strand breaks (SSBs) and double-strand breaks (DSBs) (Goldstein and Kastan, 2015). The efficacy of radiotherapy depends on its ability to induce lethal DNA damage in cancer cells and the ability of cells to repair radiation-induced DNA damage (Schaue and McBride, 2005; Buckley et al., 2020). Unrepaired and inaccurately repaired DNA double-strand breaks are the main cause of radiation-induced cell death (Morgan and Lawrence, 2015). There are two main pathways for DSB repair, nonhomologous end-joining (NHEJ) and homologous recombination (HR) (Scully et al., 2019). NHEJ occurs throughout the cell cycle and is the main repair pathway for G1 phase DSBs (Takata et al., 1998; Zhao et al., 2017). HR uses sister chromatids as a template for repair and is therefore restricted to the S/G2 phase (Takata et al., 1998; Orthwein et al., 2015).

**FIGURE 3:**
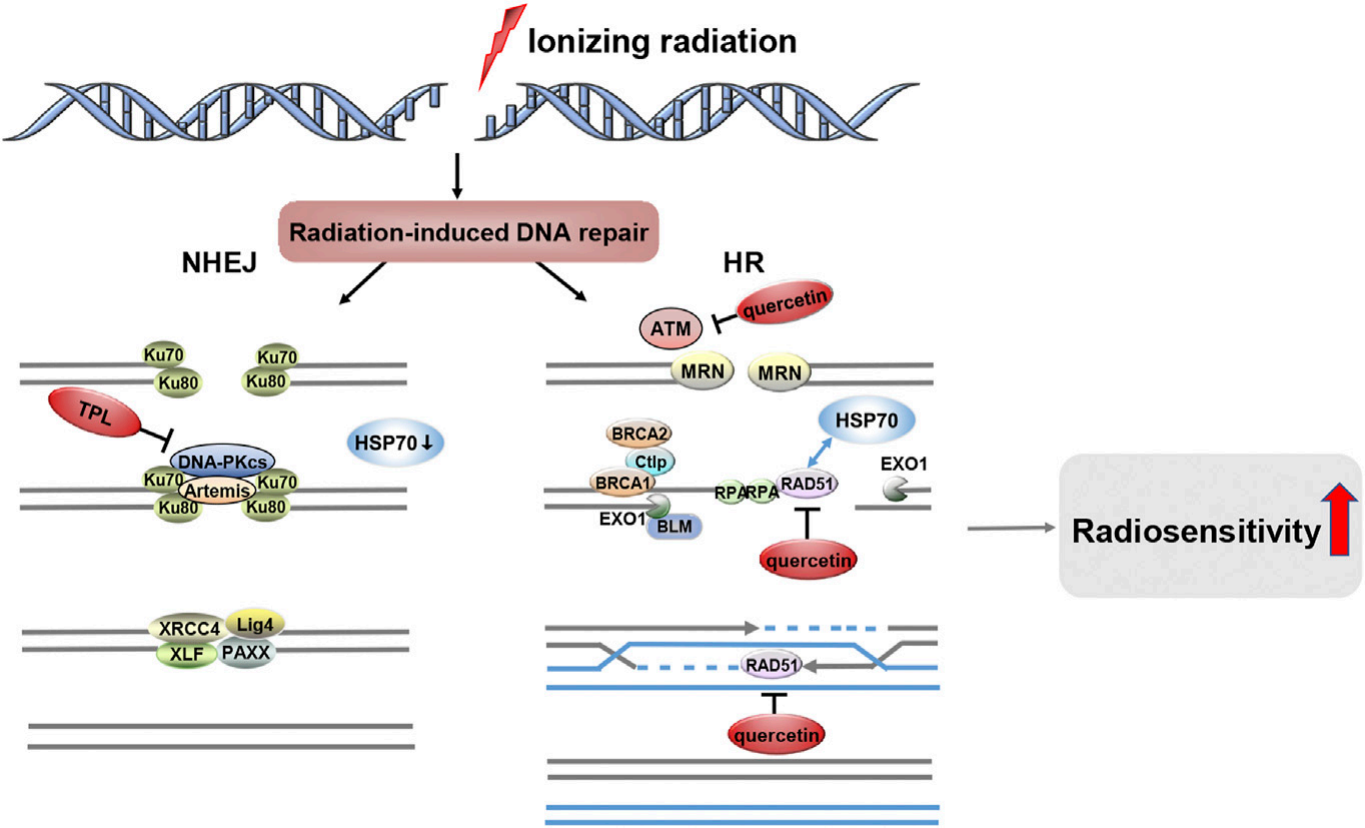
HSP70 Inhibitors Inhibit the Repair of Radiation-induced DNA Damage. After ionizing radiation-induced DSBs, two distinct DNA repair systems emerged, namely NHEJ and HR. HSP70 inhibitors such as quercetin and TPL can be involved in the DNA damage repair pathway, and they increase the radiosensitivity of tumors by inhibiting radiation-induced DNA damage repair.

Ionizing radiation increased γ-H2AX levels in a dose-dependent manner, with the abundance of γ-H2AX lesions peaking 30 min after IR and returning to baseline levels approximately 24 h after IR (Yang et al., 2020b). γ-H2AX is one of the earliest DSB-induced DNA damage repair (DDR) events (Willers et al., 2015). Several preclinical studies have shown that the combination of IR and HSP70 inhibitors exacerbates γ-H2AX lesions, increases unrepaired DSB and induces delayed DSB repair, and increases the radiosensitivity of tumor cells (van Rijn and van den Berg, 1997; Lin et al., 2012; Gong et al., 2018). Furthermore, the combination of IR and HSP70 inhibitors leads to HR defects by reducing RAD51 expression. The DNA recombinase RAD51 is the most critical component of the HR pathway, catalyzing the typical key responses of HR. In contrast, the ataxia telangiectasia mutated (ATM) kinase is an upstream regulator of RAD51 and is activated to enhance the HR response to DSB (Li and Heyer, 2008; Yang et al., 2020b). Within minutes of DSB formation, different proteins are phosphorylated by active ATM, which are required for the DNA damage response and repair (Hakem, 2008). HSP70 inhibitors can impede DNA damage repair in irradiated tumor cells, so ionizing radiation combined with HSP70 inhibitor treatment can exacerbate DNA damage and enhance radiation-induced cell death (Figure 3). *In vitro* and *in vivo* studies have confirmed that quercetin can specifically inhibit ATM activation and ATM-mediated phosphorylation of downstream targets, leading to tumor radiosensitization (Lin et al., 2012). In support of this finding, the radiosensitizing effect of quercetin was lost in ATM-deficient cells (Lin et al., 2012). Although no interaction between HSP70 and ATM was detected, HSP70 was found to interact with its downstream factor RAD51 and to be associated with radiosensitivity (Tambini et al., 2010). Gong et al. (2018) showed that quercetin reduced RAD51 expression via the p53-mediated endoplasmic reticulum stress pathway and that cells lacking RAD51 function had a defect in HR repair leading to reduced DSB repair and increased ionizing radiation-induced apoptosis. The above results highlight the potential of quercetin as a novel radiosensitizer. In addition, TPL and its derivatives exhibit cytotoxicity by inducing DNA damage, thus increasing the sensitivity of different cell types to DNA damage-based chemotherapy or radiotherapy. TPL impairs NHEJ repair by directly interacting with and inhibiting the autophosphorylation of DNA-PKcs (Cai et al., 2020). TPL can also reduce the expression of HSP70 by downregulating Sp1, thereby promoting the apoptosis of tumor cells (Arora et al., 2017). Moreover, Sp1 interacted with DNA-PKcs in several types of tumor tissues (gastric, colon, and pancreatic cancer) to participate in DNA damage repair (Odegaard et al., 1998; Hosoi et al., 2004). In conclusion, TPL can not only impair DNA repair by directly inhibiting DNA-PKcs, but may also affect the interaction between sp1 and DNA-PKcs by inhibiting DNA-PKcs, and ultimately downregulate the expression level of HSP70 to promote tumor cell apoptosis. However, the current data do not demonstrate that HSP70 inhibitors in combination with IR inhibit NHEJ activity while attenuating HR efficiency.

### Cell cycle blockade

Disturbances in cell cycle regulation play an important role in the development of tumors. The operation of the cell cycle is largely dependent on cyclin dependent kinase (CDK), which relies on the binding of cyclin to perform key functions in the orderly progression of the cell cycle. Different CDK-Cyclin complexes phosphorylate specific target proteins to stimulate cell cycle phases, facilitating the G1/S and G2/M transitions. Cell cycle arrest is a key determinant of radiosensitivity, and HSP70 causes a delay in cell growth that reduces G2/M phase arrest (Lee et al., 2001). Notably, G2/M phase cells are the most sensitive to radiation (Terasima and Tolmach, 1961; Sinclair and Morton, 1966). Lee et al. (Lee et al., 2001) examined the G2/M phase arrest in two cell lines, the vector control and HSP70-transfected cell group, by measuring the accumulation of G2/M phase cells at approximately 3, 6, 12, and 24 h after 4 Gy irradiation using flow cytometry. The cell cycle arrest was significantly reduced in HSP70 overexpressing cells at 6 and 12 h after irradiation, with a 20% and 24% reduction in G2/M phase block, respectively. These data indicate that HSP70 causes a reduction in radiation-induced G2/M phase block, resulting in reduced radiosensitivity.

In addition, inhibition of HSP70 expression significantly inhibits tumor cell growth and induces cell cycle arrest (Lin et al., 2014), so Hsp70 inhibitors could be considered to counteract the effects of Hsp70 on the cell cycle.

**FIGURE 4:**
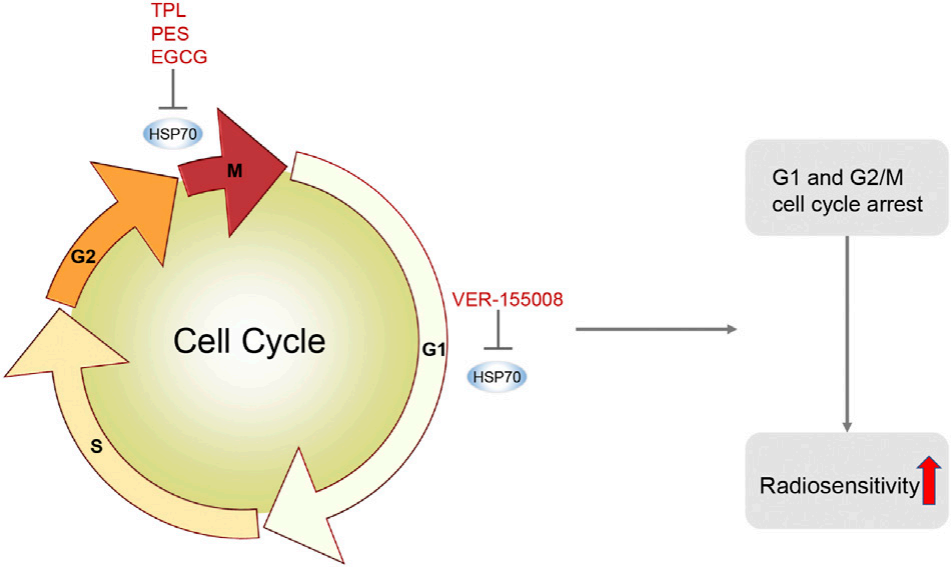
HSP70 Inhibitors Induce Cell Cycle Arrest. HSP70 inhibitors induce cell cycle arrest by inhibiting HSP70. For example, TPL induces G2/M phase arrest in nasopharyngeal carcinoma; VER-155008 induces G1 phase arrest in pleural mesothelioma; PES induces G2/M phase arrest in oral squamous cell carcinoma; EGCG induces G2/M phase arrest in MCF-7 cells in breast cancer. HSP70 inhibitors induce cell cycle arrest, make tumor cells more sensitive to radiotherapy, and increase radiotherapy-induced apoptosis.

The DNA damage caused by radiotherapy leads to the activation of cell cycle regulatory mechanisms by the relevant genes, causing cell cycle arrest at two checkpoints, the G1/S phase and the G2/M phase, and the blockage of cell cycle checkpoints is an important determinant of the radiosensitivity of tumor cells (Figure 4). HSP70 inhibitors can radiosensitize tumors by inhibiting HSP70-induced cell cycle checkpoint arrest, as in nasopharyngeal carcinoma TPL can trigger apoptosis and induce G2/M phase arrest by inhibiting GRP78, thus TPL can be used as a new radiosensitizer for the treatment of nasopharyngeal carcinoma (Li et al., 2016).

Furthermore, in pleural mesothelioma, VER-155008 inhibits HSP70 by inducing G1 cell cycle arrest and disrupting the PI3K/AKT/mTOR pathway, thereby suppressing tumor proliferation (Sakai et al., 2021). In oral squamous cell carcinoma, PES blocked the cell cycle in the G2/M phase and induced apoptosis (Jiang and Xiao, 2020). In breast cancer, EGCG induced a G2/M phase cell cycle arrest in MCF-7 cells and enhanced the radiosensitivity of breast cancer (Tran et al., 2010). In summary, HSP70 inhibitors can make tumor cells more sensitive to radiotherapy by inducing cell cycle arrest.

### Anti-tumor angiogenesis

**FIGURE 5:**
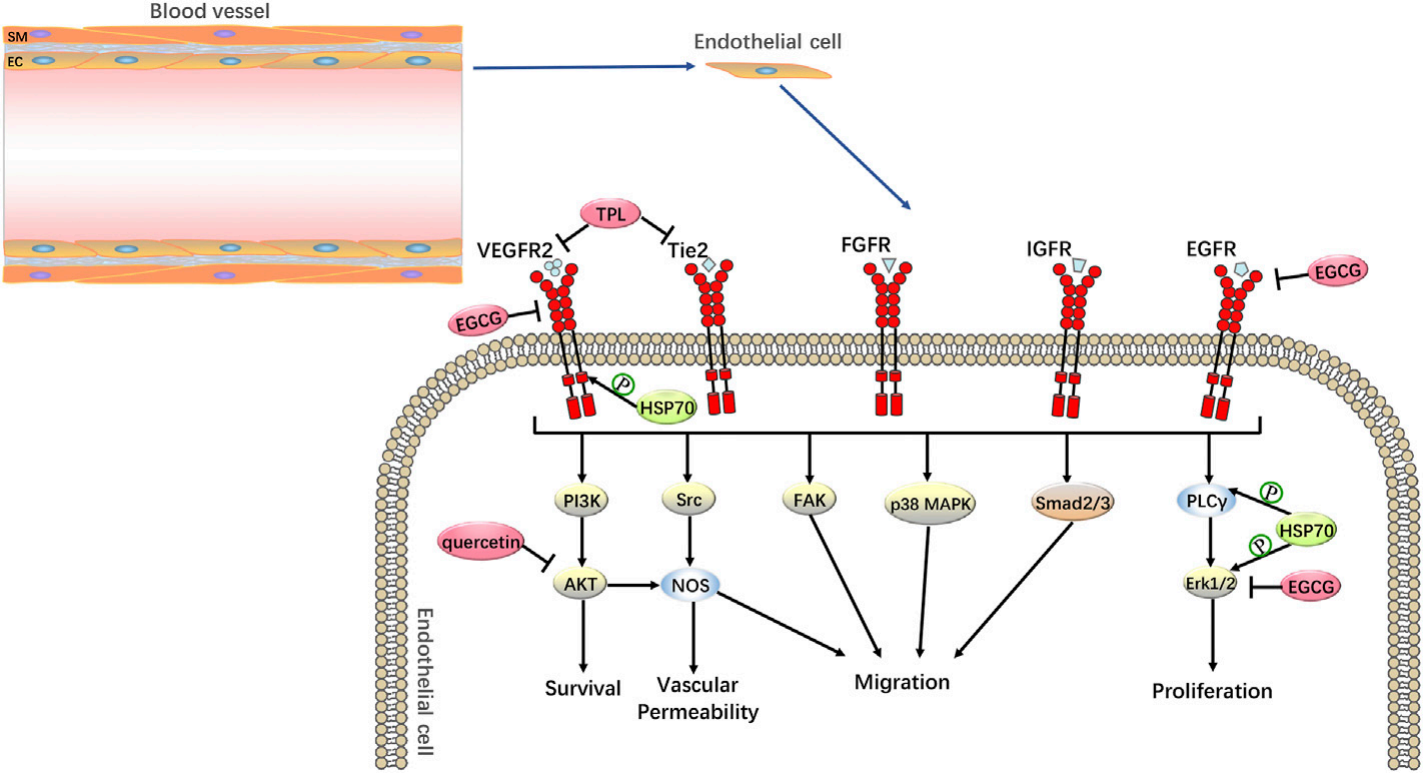
Anti-tumor Angiogenesis by HSP70 Inhibitors. HSP70 inhibitors can increase the radiosensitivity of tumor cells through anti-tumor angiogenesis. For example, in pancreatic cancer, TPL inhibits tumor angiogenesis by inhibiting Tie2 and VEGFR-2; in acute myeloid leukemia and breast cancer, quercetin targets the VEGFR-2-mediated angiogenic pathway and inhibits the expression of the downstream regulator AKT; in breast cancer, EGCG is anti-tumor angiogenic by inhibiting the expression of VEGF and suppressing the phosphorylation levels of EGFR and ERK1/2.

Solid tumors are associated with significant tumor neovascularization during their development. Angiogenesis refers to the development of new blood vessels from existing capillaries or post-capillary veins. Due to the increasing demand for oxygen and nutrients by the expanding tumor, tumor cells induce angiogenesis by secreting a variety of growth factors (Kleibeuker et al., 2012). Among them, vascular endothelial growth factor (VEGF) is an important angiogenic factor that induces endothelial cell proliferation and migration and promotes angiogenesis. VEGF not only causes reoxygenation of tumor cells, leading to excessive DNA replication and increased synthesis of radioresistant genes but also enhances tumor radioresistance by promoting the development of autophagy (Meng et al., 2014; Chen et al., 2020). Anti-VEGF can increase tumor radiosensitivity by blocking angiogenesis, inducing apoptosis in vascular endothelial cells, and activating the mTOR pathway to inhibit radiation-induced autophagy (Chen et al., 2020). Furthermore, both preclinical and clinical studies have shown that radiotherapy can affect tumour angiogenesis and that inhibition of angiogenesis can enhance the effects of radiotherapy (Kleibeuker et al., 2012). Meanwhile, Colvin et al. (2014) found that HSP70 could affect VEGF production in human breast cancer MCF-7 cells. Further studies also showed that GRP78 (HSP70) knockdown significantly inhibited VEGF-induced endothelial cell proliferation and VEGF-induced extracellular regulated protein kinase 1/2 (ERK1/2), phospholipase C-γ (PLC-γ), and VEGFR-2 phosphorylation, thereby suppressing tumour angiogenesis (Katanasaka et al., 2010). Therefore, HSP70 inhibitors can act as inhibitors of tumor-induced angiogenesis and radiosensitizers of tumor vasculature (Figure 5). One study showed that HSP70 inhibitors inhibited tumor angiogenesis and tumor metastasis (He et al., 2010). TPL inhibits pancreatic cancer tumor angiogenesis and tumor metastasis by inhibiting Tie2 and VEGFR-2 and down-regulating VEGF expression. This was confirmed by another study with quercetin, which targeted the VEGFR-2-mediated angiogenic pathway, inhibited the expression of the downstream regulator AKT, and suppressed tumor growth in acute myeloid leukemia and breast cancer (Balakrishnan et al., 2016; Shi et al., 2020). Furthermore, in breast cancer, EGCG inhibits tumor invasion and angiogenesis by suppressing VEGF expression, as well as inhibiting epidermal growth factor receptor (EGFR) and ERK1/2 phosphorylation levels, which also supports the use of HSP70 inhibitors as radiosensitizers for tumors (Khan and Mukhtar, 2010).

### Other mechanisms

The transcription factor HSF1 drives tumor development by regulating the expression of heat shock proteins, inhibiting apoptosis, promoting cancer cell proliferation and metastasis, and facilitating DNA repair (Wang et al., 2020b). HSF1 is highly expressed in a variety of tumors (Mendillo et al., 2012) and high HSF1 expression in tumor tissues is negatively correlated with the prognosis of cancer patients (Zhou et al., 2017b). For example, higher levels of HSF1 expression in breast, oral cavity, myeloma, and endometrial cancers are associated with poor prognosis and increased metastasis in patients (Santagata et al., 2011; Ishiwata et al., 2012; Engerud et al., 2014; Fok et al., 2018). In addition, gene transcription of HSP70 is also regulated by HSF1, which is activated in response to stress stimuli. Since activated HSF1 induces the expression of HSP70 and enhances cellular radioresistance (Kabakov et al., 2006), inhibition of HSF1 may be an effective way to block HSP70 expression and increase radiosensitivity (Kumar et al., 2016). Considering the role of HSF1 in tumor development, HSF1 could be a potential therapeutic target. Activation of HSF1 is a rather complex process: HSF1 binds to molecular chaperones (e.g., HSP70) to form a complex and remains free in the cytoplasm as an inactive monomer, gradually acquiring transcriptional activity in response to heat shock. First HSF1 is depolymerized from the complex and then oligomerized into a DNA-bound trimer that translocates to the nucleus. The trimeric HSF1 that enters the nucleus then binds to the heat shock element (HSE) in the promoter of the target gene. HSF1 is subsequently activated by casein kinase 2 (CK2) and calcium/calmodulin-dependent protein kinase II (CaMKII) phosphorylation, and hyperphosphorylated HSF1 acquires full transcriptional activity, thereby inducing transcription of heat shock genes (Westerheide et al., 2006; Wang et al., 2009). HSP70 inhibitors can block HSP70 expression by inhibiting HSF1, thereby reducing tumor radioresistance. Inhibition of HSF1 activation can be achieved by HSP70 inhibitors TPL and quercetin (Dai and Sampson, 2016). TPL has been found to block HSF1 activation and transcription during steps such as trimerization, phosphorylation, and DNA binding, while quercetin may inhibit HSP70 by blocking phosphorylation of HSF1 by CK2 and CaMKII (Wang et al., 2009). Tran et al. (2010) demonstrated for the first time that EGCG significantly reduced HSF1 levels in a dose-dependent manner, suggesting that EGCG can suppress the expression of HSP70 by inhibiting the expression of the HSP70 transcription factor HSF1. Furthermore, KRIBB11 was the first reported direct inhibitor of HSF1, when cells were exposed to heat shock in the presence of KRIBB11, the induction of HSF1 downstream target proteins such as HSP27 and HSP70 was blocked, inducing tumour growth arrest and apoptosis (Yoon et al., 2011). The above studies suggest that HSP70 inhibitors may also block HSP70 expression by inhibiting HSF1 to radiosensitize tumors. However, inhibition of HSF-1 not only affects the expression of heat shock proteins but also other regulatory factors involved in various cellular pathways (Fok et al., 2018). Therefore, the complex mechanisms affected by inhibition of HSF-1 should be taken into account.

HSP70 inhibitors can also induce other biological phenotypes, providing a mechanistic basis for the use of HSP70 inhibitors in combination with radiation therapy. For example, the induction of senescence, i.e., irreversible growth arrest, is considered in cancer cells as a means of stopping tumor initiation and progression (Zamin et al., 2009). Firstly, long-term exposure to HSP70 inhibitors induces a cellular senescence phenotype in many cancer cell lines, such as quercetin-mediated senescence in human colon cancer cells (Özsoy et al., 2020); TPL promotes heterochromatin formation and thus represses gene transcription by inhibiting histone H3 methylation levels and upregulating H3K9me3 and H3K27me3, ultimately inducing a senescence-like phenotype in prostate cancer cells (Tamgue and Lei, 2017); and resveratrol and quercetin significantly increase the proportion of senescence-associated β-galactosidase (SA-β-GAL)-positive cells in C6 rat gliomas (Zamin et al., 2009). All of the above studies can confirm that HSP70 inhibitors can induce events of tumor cell senescence. On the other hand, radiation therapy also triggers premature senescence in solid tumor cell lines (Mirzayans et al., 2005). This finding is attributed to the fact that IR causes massive DNA damage and activates two major DNA damage-induced signaling pathways, namely the p53/p21waf1 pathway and the P16INK4a/Rb pathway, leading to sustained cell cycle arrest and cellular senescence (Panganiban et al., 2013). In addition, it has been shown that the radiosensitivity of many cancer cells can be enhanced by promoting senescence (Nam et al., 2013). Therefore, radiotherapy and HSP70 inhibitors can combine to induce tumor cell senescence and enhance the radiosensitivity of multiple tumors, thereby inhibiting tumor progression.

In addition to the classical radiosensitization mechanisms described above, HSP70 inhibitors can also inhibit tumor growth by modulating the tumor immune response (Bandyopadhyay et al., 2008; Liang and Fu, 2008; Liu et al., 2013; Chan et al., 2017). In tumor cells, regulatory T cells (Treg) play a key role in the regulation of the tumor immune response, leading to immune tolerance through direct killer cell activity, secretion of immunosuppressive molecules, and suppression of B cells, macrophages, dendritic cells (DCs), etc (Liu et al., 2013; Tanaka and Sakaguchi, 2017). Therefore, reducing the number of Treg cells or inhibiting their function could be considered as new immunotherapeutic targets for tumor therapy. Among them, IL-10 and TGF-β, two important immunosuppressive factors produced by Treg cells, can directly kill T cells or antigen-presenting cells by secreting granzymes (Tanaka and Sakaguchi, 2017). Thus, Treg cells play a key role in tumor immune tolerance, and the HSP70 inhibitor TPL significantly reduced the proportion of Treg cells in the spleen and axillary lymph nodes of tumor-bearing mice and decreased Foxp3 levels and secretion of IL-10 and TGF-β in peripheral blood (Liu et al., 2013), these data reflect the possibility that TPL may counteract tumor immune responses and inhibit tumor growth by suppressing Treg cells and immunosuppressive factors. On the other hand, radiation therapy is an ideal tool to enhance tumor MHC-I expression, which would continuously allow a cytotoxic CD8^+^ T-cell (CTL)-mediated response to specifically kill tumor cells (Frey et al., 2017). The initiation of an immune response after radiation therapy has been demonstrated in several preclinical studies, not only because of the induction of different forms of tumor cell death, but also because the interferon-gene stimulated protein signaling pathway (STING), which senses DNA, is activated (Frey et al., 2017). Although ionizing radiation recruited Treg cells and other immunosuppressive cells into the tumor microenvironment, TPL significantly reduced the proliferation of Treg (Liu et al., 2013). Interestingly, other researchers have reported that TPL can amplify Treg cells in non-tumor cells (Zhang et al., 2009; Xu et al., 2016). These varied results suggest that the effects of natural compounds in different disease states may be complex. In addition, radiation therapy sensitizes refractory “cold” tumors to immune checkpoint inhibitors by promoting the recruitment of anti-tumor T cells (Demaria et al., 2016). In conclusion, the above data suggest that both HSP70 inhibitors and radiotherapy can improve local tumor control through an anti-tumor immune response. These potential mechanisms provide new perspectives for future exploration and offer the possibility of combining HSP70 inhibitors, radiotherapy, and immunotherapy.

## Tumor cell resistance to HSP70 inhibitor radiosensitization

With the application of HSP70 inhibitors, it is found that tumor has drug resistance to HSP70 inhibitors, which reduces their radiosensitization effect. For example, the radiosensitizing effect of the HSP70 inhibitor MAL3-101 is achieved by inhibiting HSP70ATPase activity, yet tumor cells can overcome MAL3-101-induced apoptosis through endoplasmic reticulum-associated degradation and autophagy and become resistant to HSP70 inhibitors (Moradi-Marjaneh et al., 2019).

One study found that quercetin enhanced the phosphorylation of HSP27 upon down-regulation of HSP70, and that phosphorylated HSP27 had anti-apoptotic activity, reducing the radiosensitizing effect of quercetin due to inhibition of HSP70 (Wang et al., 2009). The data reflect that inhibition of HSP70 without enhanced phosphorylation of HSP27 is beneficial in reducing the resistance of tumor cells to radiosensitization by HSP70 inhibitors, and therefore HSP70 inhibitors may be considered in combination with HSP27 inhibitors. In addition, the combination use of HSP70 inhibitors may also be an effective strategy to reduce tumor resistance to HSP70 inhibitor radiosensitization (Li et al., 2009). Quercetin down-regulated the expression of HSP70 but up-regulated the expression of GRP78 in human breast cancer cells in a dose-dependent manner. Whereas EGCG promoted quercetin-induced cell death by binding to the ATP-binding domain of GRP78 and blocking its protective function, the combined application of EGCG and quercetin inhibited both HSP70 and GRP78. The authors demonstrated a way to increase the sensitivity of breast cancer cells to a single HSP70 inhibitor by using a combination of HSP70 inhibitors (Li et al., 2009). Another study also supported the above notion that TPL enhanced kahweol-induced cytotoxicity by inhibiting Hsp70 activity and synergistically promoted colon cancer cell apoptosis (Choi et al., 2015). In summary, when heat shock protein inhibitors are used in combination, they may reduce the resistance of tumor cells to radiosensitization by HSP70 inhibitors. However, considering that toxicity to normal cells/tissues should be evaluated, their combined use still needs further study.

## Effect of dual targeting of HSP70 and HSP90 on sensitivity to radiotherapy

Many HSP90 client proteins are components of cancer cell-associated signaling pathways that ensure unlimited tumor growth and their resistance to radiotherapy. Following Hsp90 inhibition, these client proteins are degraded, disrupting multiple pathways critical for tumor cell survival. Thus, HSP90 inhibitors may also be used as agents to sensitize human tumors to radiotherapy (Kabakov et al., 2010). The majority of tumors and cancer cell lines are radioresistant to HSP90 inhibitors, therefore, there is an urgent need for a biomarker to predict the response of target cells to inhibitory therapy. HSP70 is a co-chaperone protein whose upregulation serves as a biomarker for HSP90 inhibition (Spiegelberg et al., 2020; Naz et al., 2021). In their study of the effect of HSP90 inhibitors on cellular radiation response, Kudryavtsev et al. (Kudryavtsev et al., 2017) found that radiosensitizing effects were present only in cell cultures with early and significant induction of HSP70 after treatment with HSP90 inhibitors, and that the level of HSP70 induction was positively correlated with the degree of radiosensitization. This study supports the idea that HSP70 induction can be a valid marker for predicting the effects of HSP90-inhibited radiosensitizers on tumor and normal tissue ionizing radiation. HSP70 can be used as a biomarker to quantify the inhibitory effect of low concentrations of HSP90 inhibitors on HSP90 (Mehta et al., 2020). The authors demonstrate that the inhibition of Hsp90 by even low-dose Hsp90 inhibitors can be monitored by measuring HSP70 expression in peripheral blood individual nucleated cells, which also suggests the potential use of HSP70 from blood cells as a clinical biomarker in future studies. In addition, Hsp70 as a biomarker has the advantage of being easily detected by Western blot (Kudryavtsev et al., 2017) and consistent in clinical trials (Zhang et al., 2006).

More importantly, HSP90 inhibitors have been shown to increase the radiosensitivity of tumor cells, but at the same time they also strongly enhance the expression of cytoprotective HSP70 (Ambati et al., 2014). This result is thought to reduce the overall anti-tumor effect of these compounds, and therefore dual targeting of HSP90 and HSP70 is an emerging concept for making tumor cells more sensitive to radiotherapy. Several preclinical studies have investigated the radiosensitivity of tumor cells in combined HSP90 and HSP70 therapy. Schilling et al. (2017) found that HSP70 inhibition peptide aptamer A17 inhibition of HSP70 by itself did not significantly increase the radiosensitization of lung and breast cancer cells, but it could enhance the radiosensitization of tumors by the Hsp90 inhibitor NVP-AUY922. In previous work, the research group has reported similar findings that NZ28 in combination with NVP-AUY922 achieved the same radiosensitization as high-concentration NZ28 monotherapy even when the NZ28 concentration was reduced to 1/10-1/20 (Schilling et al., 2015). Another study supports the idea that HSP90 inhibitors and HSP70 inhibitors in combination can help overcome the high radiation resistance of some tumors and sensitize them to radiation therapy (Kudryavtsev et al., 2017).

## Conclusion

HSP70 inhibitors play an important role in increasing the sensitivity of tumor radiotherapy. Important data suggest that HSP70 inhibitors have radiosensitizing effects by disrupting the activity of HSP70 client proteins, blocking multiple HSP70-dependent signaling pathways, inhibiting the repair of radiation-induced DNA damage, inhibiting cell cycle checkpoint activation, and inhibiting tumor angiogenesis. Furthermore, HSP70 inhibitors prevent HSF1 activation and transcription, induce cellular senescence and promote antitumor immunity, which may be a potential mechanism for clinical radiosensitization. As tumors are drug-resistant to HSP70 inhibitors, this can reduce the radiosensitizing effect of the inhibitor. Although several preclinical studies have demonstrated that combinations between heat shock protein inhibitors can reduce tumor resistance to HSP70 inhibitor radiosensitization, the feasibility of such regimens needs to be further investigated in the future. Currently, drugs that have been studied using heat shock proteins as targets are mainly HSP90 inhibitors, but HSP70 is a more suitable drug target than HSP90 in terms of therapeutic efficacy (Balaburski et al., 2013). However, the research on HSP70 is still inadequate and the experimental data is insufficient. Moreover, most of the experiments had a limitation, that is, they did not knock out the endpoints affected by HSP70 and then tested whether HSP70 inhibitors had the same effect on the intrinsic radiosensitivity under the same conditions, thus providing a clearer illustration of the interaction between radiosensitization and Hsp70 inhibition.

In conclusion, our data provide a strong rationale for the use of HSP70 inhibitors as radiosensitizers. It is hoped that in the near future clinically available HSP70 inhibitors, either as monoclonal antibodies or in combination with radiotherapy, will be developed as an effective route of anti-tumor therapy.
